# Exploring the feasibility and synergistic value of the One Health approach in clinical research: protocol for a prospective observational study of diagnostic pathways in human and canine patients with suspected urinary tract infection

**DOI:** 10.1186/s40814-015-0036-9

**Published:** 2015-11-10

**Authors:** Gloria Cordoba, Tina Møller Sørensen, Anne Holm, Charlotte Reinhard Bjørnvad, Lars Bjerrum, Lisbeth Rem Jessen

**Affiliations:** 1 Department of Public Health, The Research Unit for General Practice and Section of General Practice, University of Copenhagen, Øster Farimagsgade 5, P. O. Box 2099, DK-1440 Copenhagen K, Denmark; 2 Department of Veterinary Clinical & Animal Sciences, University of Copenhagen, Copenhagen, Denmark

**Keywords:** Dysuria, Microbiology, Point-of-care systems

## Abstract

**Background:**

The One Health approach is emerging in response to the development of bacterial resistance. To the best of our knowledge, the possibility to use this approach in a clinical context has not yet been explored. Thus, in this paper, we report the procedures to implement a prospective observational study of diagnostic pathways in human and canine patients with suspected urinary tract infection as a means to assess the feasibility and synergistic value of setting up One Health clinical research projects and interventions.

**Methods/design:**

A prospective observational study will compare different diagnostic pathways (i.e., 16 possible combinations of diagnostic tools) to gold standard in human and veterinary primary care practice in Denmark. Fifty primary care practices and 100 veterinary clinics will each consecutively include 20 human patients or 8–10 dogs, respectively. Data will be collected at practice and patient level comprising (a) information about the organization of the practice and access to different diagnostic tools, (b) information about clinical history, diagnostic path and treatment during the index consultation, (c) information about severity of symptoms during the 7–10 days following inclusion, and (d) urine culture (type of microorganism and susceptibility test). The feasibility and synergistic value of conducting future research, and/or designing common interventions, will be assessed by evaluating the comparability of human primary care and veterinary primary care with respect to study implementation and study results.

**Discussion:**

Results from this study will give an insight into the feasibility and synergistic value of setting-up One Health research projects in a clinical context. This is crucial if we are to embrace the One Health approach, as a legitimate strategy to implement common interventions aimed at influencing the diagnostic process in human and canine patients in order to decrease inappropriate use of antibiotics.

**Trial registration:**

The study in humans has been registered in ClinicalTrials.gov NCT02249273.

**Electronic supplementary material:**

The online version of this article (doi:10.1186/s40814-015-0036-9) contains supplementary material, which is available to authorized users.

## Background

Animals and humans share a common environment and may thereby impact each other’s health. One Health is defined as the collaborative effort of multiple disciplines to attain optimal health for people, animals, and the environment [1] by studying and controlling the risks factors that can originate diseases at the confluence of humans, animals, and their interacting environment.

For many years, this approach was only used in the area of Translational Science [2] (i.e., animal models for testing new medicaments). Nonetheless during the last 15 years, the One Health approach has gained recognition about its effectiveness for mitigating and increasing the understanding of the mechanisms involved in the spread of infectious diseases [3, 4]. Consequently, this approach has been mostly developed in the public health area regarding management of zoonotic diseases [5] and surveillance of emerging diseases [6].

Now, new routes have started to be explored as a means to take not only advantage of the potential synergism of the similarities between species but also advantage of the similarities regarding the required skills needed by medical doctors, veterinarians, and Public Health professionals, when dealing with infectious diseases [7].

In that sense, the uncontainable development of antibiotic resistance in humans and animals [8, 9] has exposed the fragility of the species barrier [10], revealing the need to promote a cross-sectorial collaboration that follows a One Health strategy, not only at a public health level but also in a clinical context.

Inappropriate use of antibiotics is one of the most important determinants for the development of antibiotic-resistant bacterial strains [11]. Consequently, during the past two decades, several studies have tried to identify the determinants of antibiotic prescription in humans and animals [12–14]. The overall conclusion is that a variety of factors influence the final decision to prescribe antibiotics, including characteristics of the patient, the prescriber, the health system, and the society.

Previous studies have shown that uncertainty about the bacterial or viral origin of the symptoms [15] and risk-avoidance attitudes [16] are associated with inappropriate prescribing of antibiotics, implying that use of accurate diagnostic tools is crucial during the decision-making process of antibiotic prescription.

Urinary tract infection (UTI) is a frequent reason to prescribe antibiotics, both in humans at primary care level [17] and in companion animals [18], fuelling the development of antibiotic-resistant strains, specifically in gram-negative rods such as extended spectrum beta-lactamase (ESBL)-producing *Escherichia coli* [19].

General practitioners (GPs) and companion animal veterinarians (CAVs) face two common challenges: (a) lack of consensus regarding the validity of different diagnostic approaches in human and canine patients with suspected UTI and, (b) lack of evidence regarding the impact of the use of these diagnostic approaches on the clinical and microbiological recovery.

The lack of consensus and evidence is highlighted in the current international recommendations regarding treatment of UTI in humans and companion animals, which tend to focus on issues of antibiotic treatment (drug selection, dose, duration, and route of administration) with less emphasis on the diagnostic process [18, 20].

Several diagnostic tools are available in human and veterinary practice to identify patients with UTI, but there is no consensus about the added value of using different diagnostic pathways. For example, some studies of human patients with uncomplicated UTI have demonstrated that symptoms alone lead to overuse of antibiotics [21–23], while other studies have concluded that the combination of specific symptoms may justify the empirical prescription of antibiotics without a need for further testing [24, 25].

Furthermore, studies assessing the validity of microscopy show wide variation in sensitivity (60–100 %) and specificity (49–100 %) to predict significant bacteriuria in humans [26, 27] and in companion animals [28–29]. There are no studies comparing microscopy with more recent diagnostic tools such as point-of-care culture and susceptibility testing in human and veterinary primary care practice.

In this paper, we describe the procedures for conducting a prospective observational diagnostic study in human and canine patients with suspected UTI. The aims of the study are (i) to assess the impact of the diagnostic process on proper use of antibiotics in human and canine patients with suspected UTI, (ii) to assess the feasibility (i.e., degree of comparability of the implementation phase), and (iii) the synergistic value (i.e., degree of comparability of the results) of the One Health approach in order to design common future interventions and research.

## Methods/design

### Design and setting

The study is designed as a prospective observational study comparing different diagnostic strategies with gold standard (culture and susceptibility testing at a reference microbiological laboratory) in human and veterinary primary care practice in Denmark.

There are five common diagnostic tests, which can be used in humans and animals with suspected UTI (Additional file 1). The interpretation of these tests can vary due to differences in the prevalence of some microorganisms, the urine collection technique, and cut-off point for significant bacteriuria on positive cultures (Additional file 2).

**Fig. 1:**
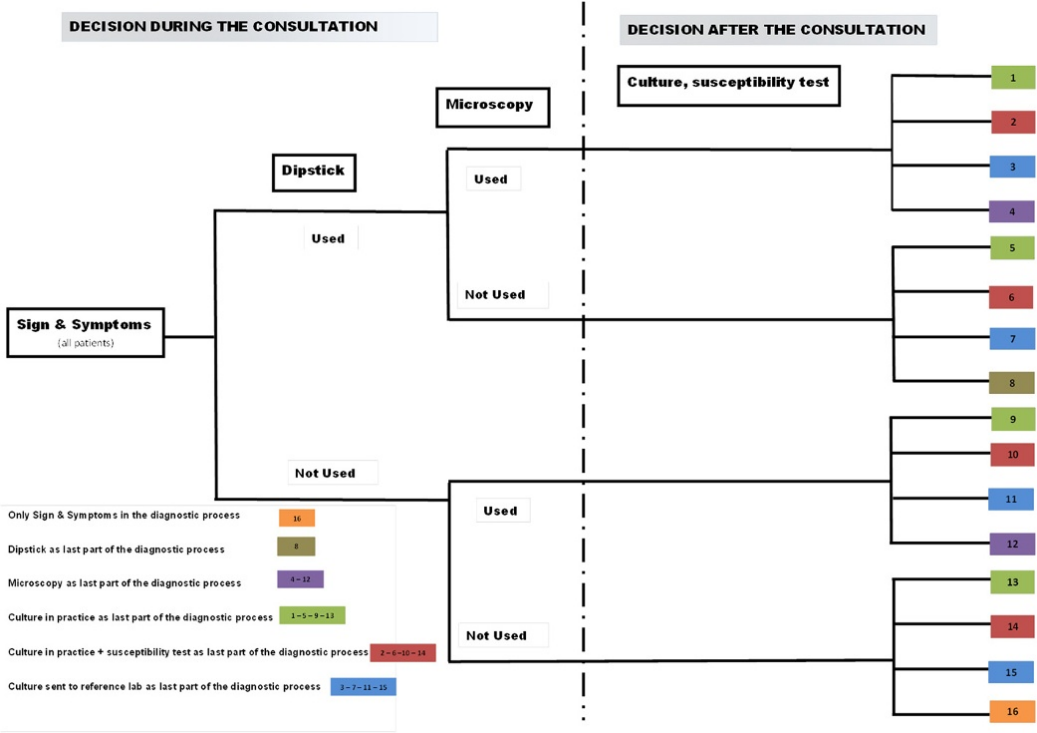
Diagnostic path in patients with suspected UTI in human and veterinary primary care practices in Denmark. The decision tree illustrates the different diagnostic pathways that can be taken during the diagnostic process of a patient with a suspected UTI. The diagnostic pathways are divided into those pathways in which the result is available during the consultation (signs and symptoms, dipsticks, microscopy) and those in which the result is available 1–3 days after the consultation (culture and susceptibility test in practice, culture and susceptibility test at a reference microbiology laboratory)

The clinical use of these diagnostic tools corresponds to 16 potential diagnostic pathways that are summarized in Fig. 1 and are mainly divided into those pathways in which the result is available during the consultation (signs and symptoms, dipsticks, microscopy) or those in which the result is available 1–3 days after the consultation (culture and susceptibility test in practice, culture and susceptibility test at a reference microbiology laboratory).

### Population

Fifty practices in the capital region and 100 veterinary clinics in Denmark will consecutively include minimum 20 human patients or 8–10 dogs, respectively. The inclusion criteria are summarized in Table 1.

**Table 1:** Summary of the inclusion and exclusion criteria for patients presenting with signs of urinary tract infection

	General practice	Veterinary practice
Inclusion criteria	≥18 years of age	Dogs of all ages
	Acute dysuria and/or frequency	Acute dysuria, frequency, hematuria, strangury, and/or malodorous urine
	Patient consulting during office hours	
	A suspected UTI	A suspected UTI
	Patient signs written informed consent	Owner signs written informed consent
Exclusion criteria	Currently taking antibiotics	Antibiotic treatment in the last 3 weeks
	Inability to fill in the symptom diary	Systemic illness
	Inability to provide a urine sample	Known chronic disease(s)
	Inability to sign an inform consent	Chronic, recurrent or relapsing UTI (three times or more in a year)
	Previous participation in this study	Inability to collect a urine sample
		Previous participation in this study

*UTI* urinary tract infection

The study is planned to last 2 years. Every 6 months, general practitioners from the capital region and veterinarians from the whole country will receive a personal invitation letter to participate until we get the expected number of professionals.

### Data collection

**Fig. 2:**
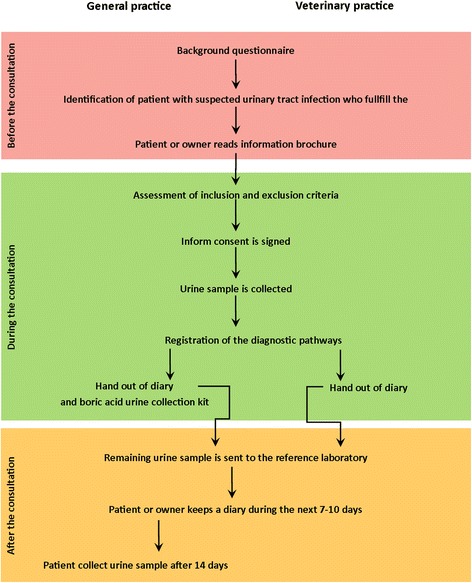
Data collection flow chart. The diagram shows the phases and milestones of data collection before, during, and after the index consultation in human and veterinary primary care practices

Figure 2 summarizes the data collection process.

Before enrollment of patients, the contact person at each practice/veterinary clinic (i.e., general practitioner, nurse, and veterinarian) will complete a background questionnaire about the organization of the practice/clinic and the access to different diagnostic tools.

On the day of the index consultation, the clinical history, diagnostic path, treatment, and other relevant decisions made during the consultation will be registered. Health care providers will consecutively include patients that fulfill inclusion criteria. Age and gender of human patients that fulfill the inclusion criteria but refuse to participate in the study will be registered.

The patient or the dog owner will keep a diary during the 7–10 days following inclusion. Human patients will be asked about potential risk factors for harboring resistant bacteria, specifically ESBL-resistant *E. coli* [30]. Both human patients and dog owners will register severity of clinical symptoms daily during 7–10 days. The validation of the severity score in humans will be published elsewhere.

Automatic text message reminders will be sent to the patient or dog owner to remind them to fill out and return the diary and to remember to send in a second urine sample for culture 2 weeks after the index consultation.

For those patients attending general practice, further information about co-morbidities, hospitalizations, and use of antibiotics will be collected from The Danish National Patient Register and The Danish National Prescription Register.

#### Urine samples

A urine sample will be collected from each patient and dog and used for two purposes: (a) gold standard culture and susceptibility testing at a reference laboratory and (b) on-site diagnostic procedures (i.e., sign and symptoms, sticks, microscopy, culture and susceptibility test in the practice/clinic). Human patients will deliver a second urine sample for culture 14 days after the index consultation in order to evaluate the microbiological effect of the treatment.

#### Gold standard

Urine samples will be sent by certified mail to the reference laboratories. For general practice, urine samples will be analyzed at the National Center for Surveillance, Prevention and Control of Infectious Diseases—Statens Serum Institute (SSI)—and urine samples from veterinary practices will be analyzed at the Department of Veterinary Disease Biology, University of Copenhagen (SUND VET DIAGNOSTIK (SVD)). Mid-stream urine and catheter-collected urine samples will be sent in boric acid preservation tubes (10 mL Sarstedt monovette and BD Vacutainer C&S Boric Acid Kit), and urine sampled by cystocentesis will be sent in plain sterile vacutainer tubes. Laboratory staff members processing and analyzing the cultures will not have access to any clinical information.

At SSI, aerobic urine culture will be performed with 1 μL on blood agar plate and “blue” agar plate (SSI Diagnostics, Denmark) and 100 μL on ESBL chromogenic culture media (Brilliance ESBL AGAR; Oxoid, UK).

ESBL plates will be examined after 1 day of incubation and read according to the color chart provided by the manufacturer. Phenotypic confirmation of ESBL production is performed by the Total ESBL Confirm Kit 98014 (Rosco Diagnostics).

Susceptibility testing will be performed and interpreted according to EUCAST standards [31] on Mueller-Hinton agar plates using Neo-Sensitabs (Sulfamethoxazole, trimethoprim, ampicillin, amoxicillin-clavulanic acid, cefpodoxime, ciprofloxacin, and nitrofurantoin, Rosco Diagnostics).

At SVD, samples will be cultured on bovine blood agar plates as well as MacConkey agar plates. Each half of the bovine blood agar plates will be prepared with sterile loops containing 1 μL and 10 μL urine, respectively. The MacConkey agar plates will be prepared with sterile loops containing 1 μL of each urine sample. All plates will be incubated aerobically overnight at 37 °C.

When reading the plates, any growth on the MacConkey plate will be noted. If growth is observed on the bovine blood agar plates, the colonies will be inspected and the number of colony-forming units (CFU) per milliliter of urine will be counted and noted. If more than one type of colony is present, each type will be subcultured on a separate bovine blood agar plate and incubated overnight at 37 °C before reading the next morning. Each type of colony will be identified to species level by matrix-assisted laser desorption/ionization time of flight (MALDI-TOF) mass spectrometry (Vitek MS RUO, France). Antimicrobial susceptibility will be tested by broth microdilution (Sensititre® COMPAN1F; TREK Diagnostic System Ltd., West Sussex, UK) according to the Clinical and Laboratory Standards Institute (CLSI) [32].

While the study is ongoing, GPs and veterinarians will not receive the results from the reference laboratories in order to avoid impacting the diagnostic procedures and case management (review bias).

### Outcomes

The outcomes and source of data related to the diagnostic pathways are presented in Table 2.

**Table 2:** Primary and secondary outcomes with data sources to investigate the impact of diagnostic and treatment procedures in patients with suspected UTI in human and veterinary primary care practices in Denmark

General practice		Veterinary practice	
Primary outcome	Source of data	Primary outcome	Source of data
Proportion of (i) appropriate decisions to treat with antibiotics and (ii) appropriate choices of antibiotic for each diagnostic path	Case report form	Proportion of (i) appropriate decisions to treat with antibiotics and (ii) appropriate choices of antibiotic for each diagnostic path	Case report form
Difference in the percentage of patients with appropriate antibiotic treatment when comparing diagnostic pathways during the consultation and diagnostic pathways after the consultation	Culture report from SSI^a^	Difference in the percentage of patients with appropriate antibiotic treatment when comparing diagnostic pathways during the consultation and diagnostic pathways after the consultation	Culture report from SVD^a^
Secondary outcomes		Secondary outcomes	
Validity of each diagnostic path	Case report form	Validity of each diagnostic path	Case report form
	Culture report from SSI		Culture report from SVD
Number of days until clinical cure (i.e., first day without symptoms from the urinary tract)	Symptom diary	Number of days until clinical cure (i.e., first day without clinical signs from the urinary tract	Symptom diary
Prevalence of uro-pathogens	Culture report from SSI	Prevalence of uro-pathogens	Culture report from SVD
Susceptibility patterns for each bacterial strain	Culture report from SSI	Susceptibility patterns for each bacterial strain	Culture report from SVD
Prevalence of ESBL-resistant *E. coli*	Culture report from SSI	Prevalence of multi-resistant bacterial strains	Culture report from SVD
Risk factors for harboring ESBL-resistant *E. coli*	Culture report from SSI	n.a.	n.a.
	Patient questionnaire		

*SSI* Statens Serum Institute, *SVD* SUND VET DIAGNOSTIK, *ESBL* extended spectrum beta-lactamase, *n.a.* not available

^a^Reference laboratories

The feasibility and synergistic value of conducting future research, and/or designing common interventions, will be assessed by evaluating the comparability of human primary care and veterinary primary care with respect to (i) the study implementation and (ii) the study results. Feasibility and synergistic value outcomes are summarized in Table 3.

**Table 3:** Outcomes to assess the feasibility and synergistic value of the One Health approach in human and veterinary primary care practices in Denmark

	Outcome
Feasibility	• Number of clinicians recruited
	• Number of patients recruited
	• Recruitment speed rate of patients
	• Number of patients followed-up
	• Proportion of data completion
Synergistic value	• Proportion of appropriate decisions to treat with antibiotics for each diagnostic path
	• Proportion of appropriate choices of antibiotic for each diagnostic path

#### Sample size calculation

**Fig. 3:**
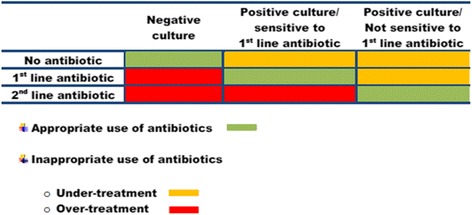
Definition of appropriate and inappropriate use of antibiotics. Appropriate use of antibiotics means that the decision about not giving antibiotics is correct as far as the culture is negative or the bacteria is susceptible to the prescribed antibiotic. Inappropriate use of antibiotics can lead to two scenarios: (**a**) under-treatment: a patient with a positive culture is not given antibiotics or the bacteria are not susceptible to the prescribed antibiotic and (**b**) over-treatment: a patient with a negative culture is given antibiotics or is unnecessarily treated with a second-line antibiotic

Due to the observational design of the study, the distribution of the diagnostic pathways is currently unknown. Nonetheless, the calculated sample sizes are based on the following assumptions: (a) the four diagnostic pathways that influence the decision during the consultation (i.e., sign and symptoms, sticks, microscopy—Fig. 1) are used in 60 % of the human patients and 80 % of the dogs, (b) a correct decision (i.e., appropriate use of antibiotics—Fig. 3) is made for 60 % of the human patients and 55 % of the dogs, while (c) an incorrect decision during the consultation is made for 40 % of the human patients and 45 % of the dogs in which any of the remaining 12 diagnostic pathways (involving culture and/or susceptibility testing) are performed. Intra-class correlation = 0.2, *α* = 0.05 and *β* = 0.2. Based on these assumptions, 900 patients from 50 practices and 800 dogs from 100 veterinary clinics are required.

### Data management

Two different databases will be created to store data from general practice and data from veterinary clinics, respectively. The data from general practice will be stored in an encrypted drive as it contains the personal identification number from each patient (CPR number) and the national identification number at practice level (ydernummer). All data will be typed twice and will be screened for data entry errors and extreme values using tables, plots, and specific commands using SAS software, Version 9.3 of the SAS System for Windows 7; copyright (c) 2002–2010 by SAS Institute Inc., Cary, NC, USA.

#### Statistical analysis

The result from the “gold standard” will provide information to assess proper use of antibiotics as described in Fig. 3. First- and second-line antibiotics for the treatment of UTI in humans and dogs are presented in Table 4*.*

**Table 4:** National Danish UTI treatment recommendations

	General practice [38]	Veterinary practice [39]
Acute uncomplicated cystitis		
First-line antibiotics	* Sulfamethizol 1 g × 2 for 3 days	* Amoxicillin 10–15 mg/kg, PO, BID–TID ≤7 days
	* Pivmecillinam 400 mg × 3 for 3 days	* Sulfa/TMP 15 mg/kg, PO, BID ≤7 days
Second-line antibiotics	* Trimethoprim 200 mg × 2 for 3 days	* Amoxicillin/clavulanic acid 12.5–25 mg/kg, PO, BID–TID ≤7 days
	* Nitrofurantoin 50 mg × 4 for 3 days	* Enrofloxacin 5 mg/kg IM/SC/PO, SID ≤7 days
Acute complicated cystitis		
First-line antibiotics	* Sulfamethizol 1 g × 2 for 3 days	* Amoxicillin 10–15 mg/kg, PO, BID–TID 7 days to 4 weeks
	* Pivmecillinam 400 mg × 3 for 3 days	* Sulfa/TMP 15 mg/kg, PO, BID 7 days to 4 weeks
Second-line antibiotics	* Trimethoprim 200 mg × 2 for 3 days	* Amoxicillin/clavulanic acid 12.5–20 mg/kg, PO, BID–TID 7 days to 4 weeks
	* Nitrofurantoin 50 mg × 4 for 3 days	* Enrofloxacin 5 mg/kg IM/SC/PO, SID 7 days to 4 weeks
Acute pyelonephritis		
First-line antibiotics	* Pivmecillinam 400 mg × 3 for 3 days	* Amoxicillin/clavulanic acid 12.5–25 mg/kg IM/SC/PO, BID–TID 4–6 weeks
Second-line antibiotics	* Ciprofloxacin 500 mg × 2 for 10 days	* Enrofloxacin 5–20 mg/kg IM/SC/PO, SID 4–6 weeks

Sensitivity, specificity, likelihood ratios, and receiver-operating characteristic (ROC) curves will be used to assess the accuracy of the diagnostic pathways.

A hierarchical logistic regression model will be constructed to assess the association between the use of different diagnostic pathways and proper use of antibiotics, while taking into consideration characteristics at patient and clinic level. The propensity score matching technique will be employed to adjust for pre-test imbalances in the different groups of diagnostic approaches.

Descriptive statistics looking at differences in feasibility and synergistic value outcomes between the medical and veterinary study groups will be presented. A qualitative assessment of the extent of comparability between the veterinary and medical disciplines will be deduced, and based on this, areas for meaningful future collaboration (i.e., common educational interventions/research) will be identified.

#### Ethics and dissemination

The study does not represent any risk for the patients due to the observational design. The diagnostic process and the treatment will be registered as it is currently done in everyday practice. Consequently, the course of disease and the treatment strategies are not affected by participation or not in the study.

The Regional Ethical Committee has approved development of the biobank, and informed written consents will be obtained from all human participants and dog owners.

Results of the study will be published in medical and veterinary journals as well as in journals promoting the One Health approach. The results of the project will be presented in scientific conferences in which novel strategies to curb the development of resistant bacterial strains are discussed.

## Discussion

This study is designed to contribute with unique information concerning the feasibility and synergistic value of setting-up One Health projects in a clinical context by comparing implementation outcomes as well as the impact of the diagnostic process on proper use of antibiotics in human and canine patients with suspected UTI.

### Methodology—strengths and limitations

The most important strength of the study is the pragmatic design. Hence, it is expected to increase applicability and generalizability of the findings as it is a snap-shot of the decision-making process during daily practice.

The pragmatic design is expected to encourage the consecutive inclusion of the clinically relevant population (i.e., all symptomatic patients with suspected UTI) in order to avoid selection bias. Subgroup analyses of the performance of the diagnostic pathways are performed in order to avoid misinterpretation of the performance of these pathways due to the case-mix of patients (spectrum bias) [33].

Furthermore, we will control review bias [34] by blinding the attending clinicians to the result of the “gold standard”, while technicians assessing the gold standard are blind to the results of the diagnostic process and management. Finally, the diagnostic pathways are not used to determine the final outcome (incorporation bias).

The observational design of the study is however also an important limitation. As we do not influence the diagnostic and treatment procedures already used in the different practices, the validity of the diagnostic pathways is affected by the variation caused by inter-observer interpretation, use of different commercial brands for the different diagnostic tools, the preferences for using certain diagnostic tools, and the tendency to prescribe antibiotics. We expect to be able to quantify the impact of such variation by developing hierarchical models with random effects and performing sensitivity analysis for specific groups.

Although the study aims at recruiting a wide variety of practices and veterinary clinics, we cannot rule out that they will differ in some basic characteristics from the general practice and veterinary clinic population. Hence, when interpreting the results, we will need to assess the extent of representativeness of our sample and stress out that our results could be the most conservative scenario as previous studies have shown that health professionals participating in research studies or audits have a lower prescription pattern in comparison with their counterparts that do not participate in this type of activities [35].

Finally, in human patients, we cannot control the way patients collect the urine sample; nonetheless, there is some evidence suggesting that the collection technique is not important with regard to sample contamination [36].

### One Health approach—challenges and opportunities

The lack of validated diagnostic strategies is a challenge to both general practitioners and companion animal veterinarians, when managing patients with suspected UTI. The increasing number of antibiotics used for both animals and humans makes the One Health approach a logic alternative strategy that can bring about effective solutions. Nonetheless, it is crucial to know first to what extent GPs and CAVs can work together and the relevance of such work.

As stated by Lerner et al. [37], the philosophy of the One Health approach needs to be written. In that sense, we expect to contribute with state-of-the-art knowledge about the feasibility and synergistic value, bearing in mind that better cooperation and coordination does not mean integration.

However, the potential identification of similar challenges in the diagnostic process, opens a unique possibility to implement common interventions related to changing clinical practice behavior (i.e., use of new technologies to support the decision-making process, training to cope with uncertainty) and hopefully promote rational antibiotic use in humans and dogs for the benefits of all parts.

Finally, it is important to highlight that there are several difficulties on the road to One Health clinical projects. Lack of a broad availability of funds that support this type of initiative has been the most important challenge in order to design the study as aligned as possible. For example, the second urine sample could not be funded on the veterinary side, thus we will not obtain information about the microbiological recovery of the dogs after 2 weeks.

### Conclusion

This is the first clinical research protocol aimed at exploring the feasibility and synergistic value of using the One Health approach in a clinical context. Results from this study are crucial, if we are to embrace the One Health approach as a legitimate strategy. A strategy, in which the advantages of the inter-disciplinary work can bring about solutions to the diagnostic challenges encountered in daily practice, when making the decision of prescribing antibiotics or not. Improving the accuracy of the diagnostic process could potentially reduce the unnecessary prescription of antibiotics, which in the long-term perspective could contribute with curbing the development of resistant strains.

#### Current status

The recruitment phase has started. Thirty practices have recruited 400 patients, while 96 veterinary clinics have recruited 153 dogs.

## Additional files

Additional file 1: **Overview of available diagnostic paths.** One Health overview of available diagnostic paths for patients with suspected urinary tract infection in human and veterinary primary care practices in Denmark
Additional file 2: **Overview of factors influencing the interpretation of urine samples.** One Health overview of factors influencing the interpretation of urine samples from patients with suspected urinary tract infection in human and veterinary primary care practices in Denmark
