# Supplementary material for: Exploring the feasibility and synergistic value of the One Health approach in clinical research: protocol for a prospective observational study of diagnostic pathways in human and canine patients with suspected urinary tract infection
Source: Pilot Feasibility Stud. 2015 Nov 10;1:38. doi: 10.1186/s40814-015-0036-9 (PMC5154006; doi:10.1186/s40814-015-0036-9)
Supplement: Additional file 1: — Overview of available diagnostic paths. One Health overview of available diagnostic paths for patients with suspected urinary tract infection in human and veterinary primary care practices in Denmark [file 40814_2015_36_MOESM1_ESM.docx]

**Additional file 1.** One Health overview of available diagnostic paths for patients with suspected urinary tract infection in human and veterinary primary care practices in Denmark

|  | **General Practice** | **Veterinary clinics** |
| --- | --- | --- |
| **Sign and symptoms** | | |
| Most frequently reported | Dysuria, frequency, urgency of urination[[21](#_ENREF_21)] | Dysuria, frequency, urgency of urination[[14](#_ENREF_14)] |
| **Dipstick Analysis** | | |
| Test used for diagnosis | Nitrite, leukocyte, blood[[26](#_ENREF_26)] | Primarily blood and protein[[14](#_ENREF_14)] Nitrite and leukocyte not reliable in dogs[[27](#_ENREF_27), [28](#_ENREF_28)] |
| **Microscopy** | | |
| Criteria used for diagnosis | Bacteria quantity, morphology, motility.  Leukocyte quantity | Bacterial and leukocyte quantity and presence of intracellular bacteria |
| Type of microscope | Light and phase-contrast | Light microscopy, stained sediment |
| **Culture in practice** | | |
| Culture medium | Chromogen agar  CLED- agar  MacConkey-agar  *E.coli* agar | Chromogen agar  Blood agar  CLED- agar  MacConkey-agar  *E.coli* agar |
| **Susceptibility test in practice** | | |
| Disk diffusion test for | Sulfonamides, Mecillinam, Nitrofurantoin, Ampicillin, Ciprofloxacin, Trimethoprim | Multiple different diffusion disks (containing 32 different antibiotics) are available on the veterinary market |
| Agar dilution test | Sulfonamides, Mecillinam, Nitrofurantoin, Ampicillin, Trimethoprim | Ampicillin, Amoxicillin/Clavulanic acid, Oxacillin, Enrofloxacin, Trimethoprim/sulfonamide |
