# Supplementary material for: Exploring the feasibility and synergistic value of the One Health approach in clinical research: protocol for a prospective observational study of diagnostic pathways in human and canine patients with suspected urinary tract infection
Source: Pilot Feasibility Stud. 2015 Nov 10;1:38. doi: 10.1186/s40814-015-0036-9 (PMC5154006; doi:10.1186/s40814-015-0036-9)
Supplement: Additional file 2: — Overview of factors influencing the interpretation of urine samples. One Health overview of factors influencing the interpretation of urine samples from patients with suspected urinary tract infection in human and veterinary primary care practices in Denmark [file 40814_2015_36_MOESM2_ESM.docx]

**Additional file 2.** One Health overview of factors influencing the interpretation of urine samples from patients with suspected urinary tract infection in human and veterinary primary care practices in Denmark

|  | **General Practice**[[29](#_ENREF_29)] | **Veterinary clinics** |
| --- | --- | --- |
| **Microorganisms** | | |
| Most frequent primary pathogens° | *E.coli*  *staphylococcus saprophyticus* | *E.coli*  *Staphylococcus pseudintermedius Enterococcus spp.*  *Proteus spp.*[[30](#_ENREF_30), [31](#_ENREF_31)] |
| Most frequent Secondary pathogens^¥^ | *Klebsiella spp.*  *Enterobacter spp.*  *Proteus spp.*  *Morganella morganii*  *S. aureus*  *P. Aeruginosa*  *Enterococcus spp.* | *n.a.* |
| **Urine collection technique** | | |
|  | Mid-stream urine^*^ | Cystocentesis, catheter, mid-stream urine[[14](#_ENREF_14)] |
| **Cut-off point for significant positive culture** | | |
| Primary pathogens° | >10^3^ CFU/ml | Cystocentesis: >10^3^ CFU/ml  Catheter: > 10^4^ CFU/ml in males  > 10^5^ CFU/ml in females  Voided: > 10^5^ CFU/ml |
| Secondary pathogens^¥^ | >10^4^ CFU/ml for women  >10^3^ CFU/ml for men | Like primary pathogens |

° Species that have the ability to cause urinary tract infection in individuals with normal urinary tracts.

^¥^ People: Species that seldom cause primary infection in patients with normal urinary tracts, but often occur in hospital-acquired urinary tract infection.

n.a. Not applicable.
